# Fabrication and electromagnetic performance of talc/NiTiO_3_ composite

**DOI:** 10.1098/rsos.171083

**Published:** 2018-02-07

**Authors:** Wen-Li Qin, Tian Xia, Ying Ye, Ping-Ping Zhang

**Affiliations:** Ocean College, Zhejiang University, Zhoushan 316021, People's Republic of China

**Keywords:** talc, NiTiO_3_, electromagnetic wave absorption, reflection loss

## Abstract

In this study, the electromagnetic (EM) performance of talc/NiTiO_3_ composite was evaluated. The morphology of talc displayed a lamella structure; there were many nanoparticles of NiTiO_3_ coated on the talc lamella_._ Thermal destruction occurred, which increased the surface area from 2.51 m^2^ g^−1^ to 79.09 m^2^ g^−1^ at the calcined stage at 650°C. The presence of NiTiO_3_ increased dielectric loss and magnetic loss of talc. The calculation of EM wave absorption of talc/NiTiO_3_ obtained a maximum reflection loss of −11.94 dB at the thickness of 6.85 mm; the optimum thickness for microwave absorption is 6.3–7.3 mm. This study revealed a new approach for fabricating an EM absorber and broadening applications of both talc and NiTiO_3_ in EM absorption.

## 1. Introduction

Talc is a widely distributed layered silicate mineral [1–3] with a composite of Mg_3_Si_4_O_10_(OH)_2_. Compared with other silicate minerals, talc has good surface affinity, high chemical stability and high resistance to heat [4]. It is commonly used in the manufacture of paper, linoleum, ceramics, coatings, cosmetics, plastics and so on [5,6]. For example, Refaa *et al.* [7] melted polylactide (PLA) with talc and found that the addition of talc as a nucleating agent contributes to the crystallization kinetics without affecting the crystal structure of the PLA and lamellae thickness, which is an application of talc in polymer synthesis.

With the broadened application of electromagnetic (EM) waves in various fields, such as wireless communication or microwave heating, many drawbacks concerning EM interference and pollution have emerged [8–12]. Therefore, there is an increasing demand for EM wave absorbers in many civil and military fields, either to protect human beings from irradiation or to prevent military equipment from being detected by radar waves [13–16]. Ideal EM wave absorbers have low density, high absorption, broad bandwidth and thin thickness for EM wave absorption [15–19]. There are many studies conducted to achieve this aim, mainly focused on polymers [20], carbon [21,22], SiC [23] and ferrite [11,12,24].

In recent years, considerable attention has been paid to super-paramagnetic materials such as Ni, along with titanate (Ti) with enhanced high surface area [25–30]. With a narrow band gap of −2.18 eV, NiTiO_3_ possesses significant photocatalytic property and unique photo response in the range of visible light [31], which was usually fabricated as a photocatalyst, not EM wave absorber: Zeng *et al.* synthesized it for photocatalytic water splitting by harvesting solar energy; the rate of hydrogen generation was about threefold that of pure g-C_3_N_4_ [32]. Pugazhenthiran *et al.* [30] prepared it for photocatalytic degradation of ceftiofur sodium (CFS); nearly 97% CFS was mineralized under direct sunlight irradiation. These studies broadened the NiTiO_3_ applications in water treatment for its enhanced photocatalytic ability. Also some efforts were undertaken in the pursuit of exploring the EM performance of NiTiO_3_; Lenin *et al.* reported that iron-doped nickel titanate (Fe^3+^/NiTiO_3_) ferromagnetic nanoparticles displayed enhanced ferro-dielectric behaviour at higher Fe content [25]. However, it still focused on EM absorption of Fe, not NiTiO_3_. Nevertheless, there have been no studies reported on the EM parameters of combined talc and NiTiO_3_, which will broaden applications of both talc and NiTiO_3._

In this study, we present a straightforward method to fabricate a novel EM absorber. As the talc has amphiprotic property [33], talc sheets will be a good carrier for tetrabutyltitanate and nickel acetate to fabricate NiTiO_3_. The morphology and structure of talc/NiTiO_3_ composite were examined using XRD and SEM. The complex permittivity and permeability of talc/NiTiO_3_ composites were determined first, the reflection loss (RL) of talc/NiTiO_3_ composite for EM waves at a frequency of 1–18 GHz was calculated as a function of thickness, and the optimum thickness and microwave frequency of the composite were obtained.

## 2. Material and methods

### 2.1. Materials

Talc powders with particle sizes of 500–700 nm and chemical composition of Mg_3_Si_4_O_10_(OH)_2_ were obtained from Jiangxi Province, China. Tetrabutyltitanate (TNBT), nickel acetate (C_4_H_6_O_4_Ni·4H_2_O) and ethanol are of analytical reagent grade. They were purchased from Aladdin Industrial Co., Ltd, Shanghai, China and were used without further purification.

### 2.2. Preparation of talc/NiTiO_3_ composite

In the typical procedure, 6 g talc and 6.8 g TNBT were dissolved in 10 ml of ethanol solvent, then ground until ethanol almost volatilized. In the second step, 5.8 g of C_4_H_6_O_4_Ni·4H_2_O was added with 10 ml of ethanol solvent, which was also then ground until ethanol almost volatilized. The obtained product was calcined in a muffle (Nabertherm N7/H, Germany) at 450, 550 and 650°C for 2 h at a heating rate of 2°C min^−1^. The obtained talc/NiTiO_3_ products were labelled TN 450, TN 550 and TN 650.

### 2.3. Characterizations

X-ray diffraction (XRD) patterns of the samples were examined by a Rigaku D/Max 2550 X-ray Diffractometer (Japan) by Cu K*α* radiation (*λ* = 0.15418 nm) at 60 kV and 450 mA. The microstructures of the samples were observed using scanning electron microscopy (SEM, Hitachi, Japan) at an accelerating voltage of 3 kV. Distribution of key elements—silica (Si), nickel (Ni) and titanium (Ti)–-in talc/NiTiO_3_ composite was mapped by energy-dispersive X-ray spectroscopy (EDS). Brunauer–Emmett–Teller (BET) surface area and pore characteristics were analysed using nitrogen adsorption–desorption (AUTOSORB-IQ-MP, USA) at 77 K. The BET surface area, pore volumes and pore sizes were calculated by the software Quantachrome ASiQWin (version 3.01). The EM parameters were determined by a HP8720ES vector network analyser (Agilent, USA) using the T/R coaxial line method at an EM wave frequency of 1–18 GHz and thickness of 2 mm, using paraffin as the substrate. The filling rates were 50%. The relative complex permittivity (*ε* = *ε*′ − *jε*′′) and permeability (*μ* = *μ*′ − *jμ*′′) were calculated from the measured T/R coefficients. The measurement errors are less than 10% when *ε*′ < 15.

## 3. Results and discussion

### 3.1. X-ray diffraction

**Figure 1.**
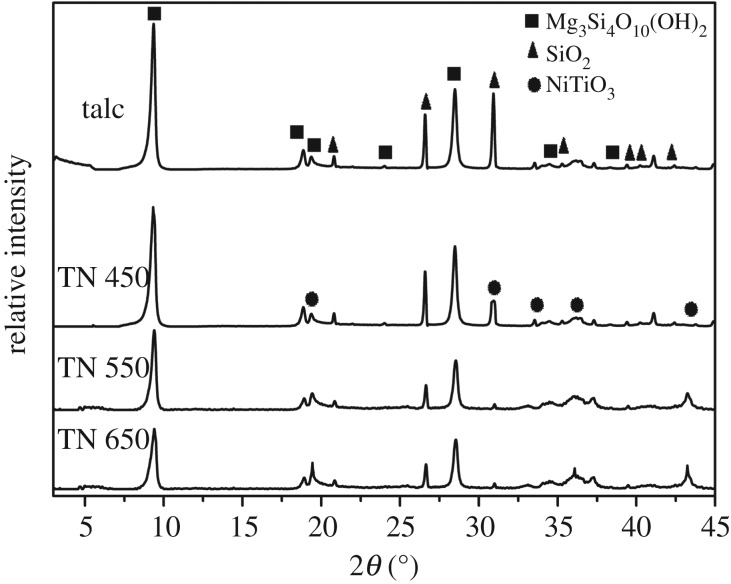
XRD patterns of talc, TN 450, TN 550 and TN 650.

Figure 1 shows the XRD patterns of talc, TN 450, TN 550 and TN 650. All samples exhibited characteristic talc peaks at 9.38° and 29.30° (Mg_3_Si_4_O_10_(OH)_2_, JCPDF # 19-0770). It is indicated that the crystal structure of talc was preserved during the preparation of the samples. The intensities of these peaks generally decrease after the talc lamella is coated with NiTiO_3_, ascribed to the decreased mass ratio of talc in the samples. With the calcined temperature increased from 450 to 650°C, the characteristic peaks of NiTiO_3_ (JCPDF # 76-0335) at 19.29° (*d_003_*), 33.12° (*d_104_*), 35.69° (*d_110_*), 38.61° (*d_015_*), 39.17° (*d_006_*) and 41.98° (*d_021_*) are enhanced in talc/NiTiO_3_ composite, which confirmed that there is a NiTiO_3_ phase in talc/NiTiO_3_ composite and higher temperature resulted in higher NiTiO_3_ content.

### 3.2. Scanning electron microscopy

**Figure 2.**
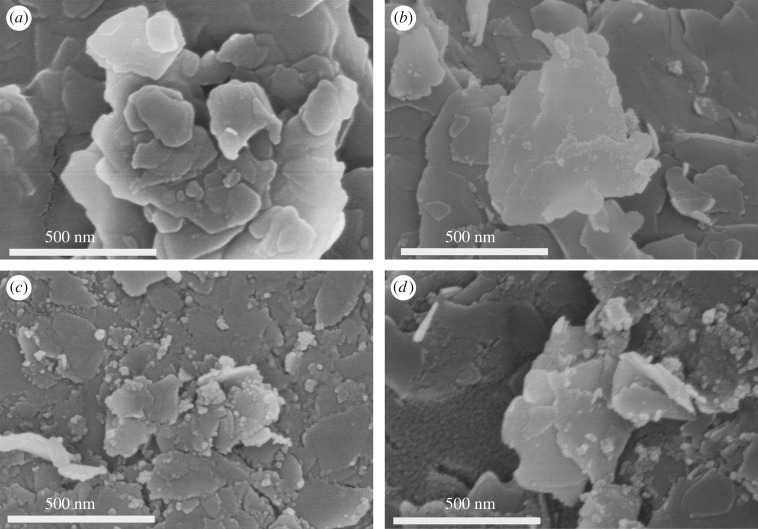
SEM images of talc (*a*), TN 450 (*b*), TN 550 (*c*) and TN 650 (*d*).

**Figure 3.**
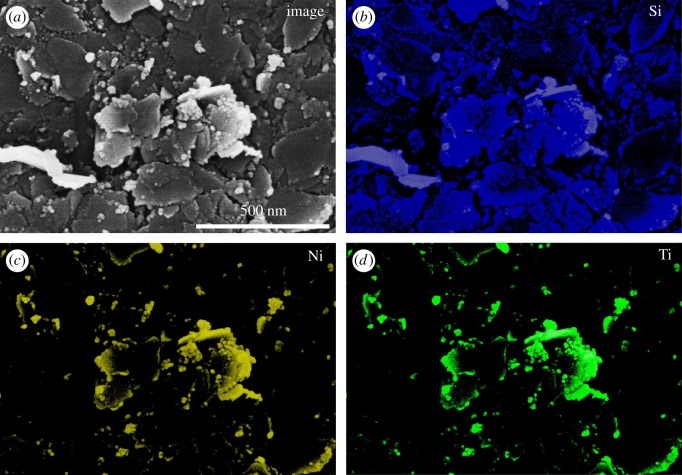
Element mapping by EDS of TN 550.

The SEM images of talc and talc/NiTiO_3_ composite are presented in figure 2. The surface feature of talc (figure 2*a*) exhibited a smooth lamella-shaped structure, and the widths of most lamellae are between 50 and 500 nm. When coated with NiTiO_3_, as shown in figure 2*b–d*, the resulting products (TN 450, TN 550 and TN 650) preserved the lamella-shaped structure, but the widths of most lamellae are less than 300 nm. This indicates that talc/NiTiO_3_ was ashed due to the occurrence of thermal destruction [34]. It also can be seen that there were many more nanoparticles of less than 20 nm covering the surface of lamellae at higher calcined temperature. Figure 3 shows the dispersion of Si, Ni and Ti in these nanoparticles by element mapping in EDS measurements using the SEM system. The constituent with the most content in talc (Mg_3_Si_4_O_10_(OH)_2_) is Si. It is found that Ni and Ti also were observed in the place of nanoparticles, indicating that NiTiO_3_ was synthesized successfully and covered the surface of lamellae, resulting in the lamellae presenting less smooth surface features than talc. It was also verified that talc sheets were a good carrier to catch TNBT and nickel acetate for fabricating NiTiO_3_.

### 3.3. Brunauer–Emmett–Teller surface area and pore analyses

**Figure 4.**
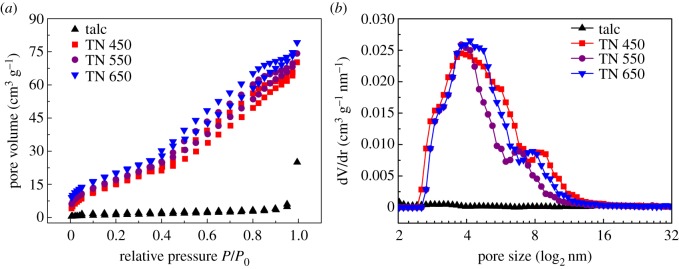
(*a*) Nitrogen adsorption and desorption curves and (*b*) pore size distribution of talc and TN 450, TN 550 and TN 650.

The BET surface area and pore size distribution may affect the EM wave absorption of talc/NiTiO_3_ composite via the scattering effect [16], smaller size will benefit the impinging of EM radiation and higher BET surface area will increase permittivity. Their pore size distributions, specific surface areas (*S*_BET_), total pore volumes (*V*_t_) and average pore diameter (*d*) were also calculated (table 1). As shown in figure 4*a*, the adsorption and desorption curves of TN 450, TN 550 and TN 650 belong to type IV isotherms for their characteristic hysteresis loops, whereas that of talc showed type I isotherms characterized by a nearly horizontal plateau and a ‘tail’ near the saturation pressure [19]. As shown in table 1 and figure 4*b*, the average pore size decreased from 34.28 nm of talc to 7.06 nm of TN 450, 6.75 nm of TN 550 and 6.67 nm of TN 650. The newly formed pores were mainly distributed in the range of between 2 and 16 nm. The BET surface area of TN 650 (79.05 m^2^ g^−1^) was much larger than that of talc (2.51 m^2^ g^−1^). The decreasing average pore size and increasing BET surface area resulted from two contradictory reasons: one is the thermal destruction that reduces the pore size [19], which occurs at higher temperatures; on the other hand, NiTiO_3_ was not only coated on the surface of talc lamellae, but also intercalated between talc layers with subsequent formation of NiTiO_3_ nanoparticles that increased porosity. Correspondingly, the total pore volume was also increased from 0.021 cm^3^ g^−1^ (talc) to 0.139 cm^3^ g^−1^ (TN 650); meanwhile the mesopore volume was also increased from 0.020 cm^3^ g^−1^ (talc) to 0.130 cm^3^ g^−1^ (TN 650) [35], indicating that a more porous structure of talc/NiTiO_3_ composite had formed.

**Table 1.** BET surface areas and pore volumes of talc, TN 450, TN 550 and TN 650.

samples	BET surface area (m^2^ g^−1^)	total pore volume (cm^3^ g^−1^)	mesopore volume (cm^3^ g^−1^)	mesopore fraction (%)	average pore size (nm)
talc	2.51	0.022	0.020	90.91	34.28
TN 450	69.21	0.098	0.94	95.92	7.06
TN 550	73.46	0.115	0.109	96.46	6.75
TN 650	79.05	0.139	0.130	93.53	6.67

### 3.4. Permittivity and permeability

**Figure 5.**
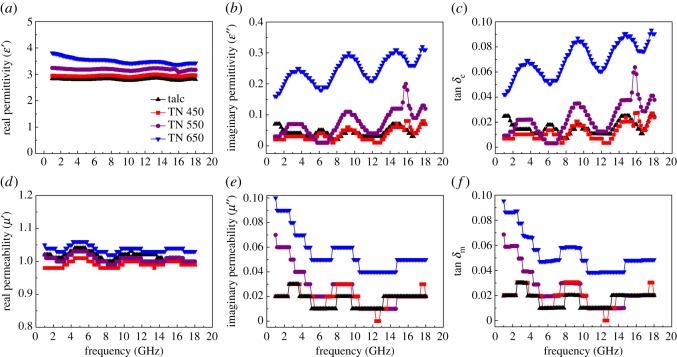
Frequency dependence of (*a*) real permittivity, (*b*) imaginary permittivity, (*c*) dielectric loss tangent, (*d*) real permeability, (*e*) imaginary permeability and (*f*) magnetic loss tangent of talc, TN 450, TN 550 and TN 650 with a volume fraction of 50% and thickness of 2 mm.

The frequency dependence of EM parameters of talc/NiTiO_3_ composite was detected using a vector network analyser in the range of 1–18 GHz. As shown in figure 5*a,b*, the real (*ε′*) and imaginary permittivities (*ε′′*) of talc are in the ranges 2.75–2.86 and 0.03–0.07, respectively. The *ε′* kept gently with the increasing of frequency, while the *ε′′* displayed some fluctuations. Consequently, their dielectric loss tangents (tan *δ*_e_ = *ε′′*/*ε′,* figure 5*c)* were generally invariant in the range of 0–0.025. When coated with NiTiO_3_, the *ε′* of talc/NiTiO_3_ increased between 2.90 and 3.00 for TN 450, 3.09 and 3.24 for TN 550, and 3.37 and 3.81 for TN 650, which are larger than that of talc. The *ε′′* of TN 450 and TN 550 increased slightly with frequency, between 0.01 and 0.19; the maximum *ε′′* of TN 650 was 0.31, also larger than that of talc. It is well known that *ε′′* *≈* *σ*/2π*ε*_0_*f* according to free electron theory [36], where *σ* is the conductivity of the sample; that is, higher *ε*′′ values require higher conductivities. The *ε′′* values of talc/NiTiO_3_ are almost increased with frequency, indicating that the conductivity of talc/NiTiO_3_ increased with frequency, which may be attributed to the eddy current effect. Moreover, the porous structure of talc/NiTiO_3_ was formed according to its BET surface area and pore characteristics. This phenomenon resulted in the formation of a more compact conductive network [34]. Consequently, the tan *δ*_e_ value of talc/NiTiO_3_ increased to the maximum value at 0.027 for TN 450, 0.064 for TN 550 and 0.091 for TN 650, where larger tan *δ*_e_ values show higher dielectric loss. This indicated that the coating of talc/NiTiO_3_ will increase the dependence of *ε′′* on frequency, which may increase the conductivity of the sample at higher temperature, resulting in talc/NiTiO_3_ composite showing higher permittivity than talc.

**Figure 6.**
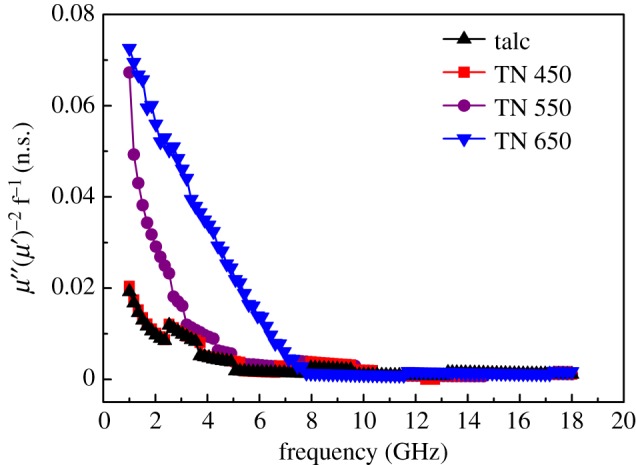
Frequency dependence of *C* = *μ*′′ (*μ*′)^−2^ *f* ^−1^ of talc, TN 450, TN 550 and TN 650.

Figure 5*d,e* shows the frequency dependence of the relative complex permeability of talc/NiTiO_3_ composite. Because of the absence of magnetic components, the real (*μ′*) and imaginary permeabilities (*μ′′*) of talc and talc/NiTiO_3_ composite are about 1.00–1.06 and 0.01–0.10, respectively. Four samples showed zigzag curves for *μ′* and *μ′′* values. Nevertheless, the *μ′* and *μ′′* values of talc/NiTiO_3_ composite were slightly higher than those of talc and TN 650 exhibited the highest value; the maximum *μ′′* value is at 1–2 GHz. As a result, the magnetic loss tangent (tan *δ*_m_ = *μ′′*/*μ′*, figure 5*f*) of talc and TN 650 is below 0.030 and 0.095, respectively. The magnetic loss of talc/NiTiO_3_ composite in the GHz region can be a result of natural resonance or eddy current. The *C = μ′′ (μ′)*^−2^*f*^−1^ is introduced to determine the magnetic loss of talc/NiTiO_3_ composite in this study [36]. As shown in figure 6, the *C* value exhibits a quicker decrease from 1 to 10 GHz at higher temperatures and maintains at about 0.001 between 10 and 18 GHz. It is indicated that the magnetic loss of talc/NiTiO_3_ composite at 10–18 GHz is dominated by the eddy current effect. The resonance peak is not found because of the small *μ′′* values and larger measurement errors. The complex permittivity and permeability values of talc/NiTiO_3_ composite suggest that the presence of NiTiO_3_ will enhance the EM wave absorption, mainly induced by dielectric loss, and higher calcined temperature results in higher performances.

### 3.5. Electromagnetic wave absorption properties

According to transmission line theory, the RL of absorbers can be evaluated from the measured complex permittivity and permeability using the following equations [37]:

3.1 $$Z_{\mathrm{in}}=Z_{0}\sqrt{\frac{\mu }{\varepsilon }}\tanh\left[j\frac{2\pi fd}{c}\sqrt{\mu \varepsilon }\right]$$

and

**Figure 7.**
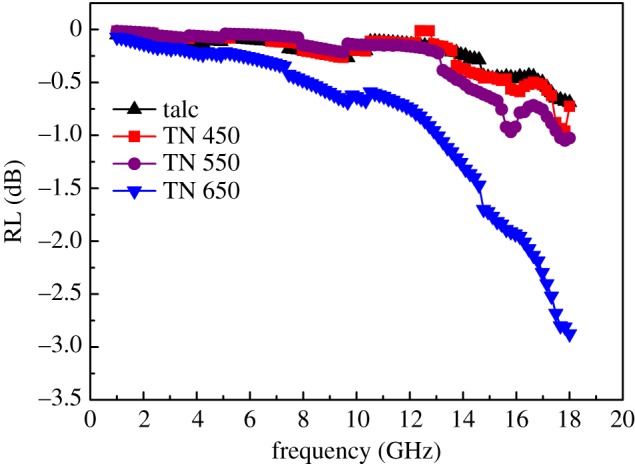
Frequency dependence of RL for talc, TN 450, TN 550 and TN 650 with a volume fraction of 50% and thickness of 2 mm.

3.2 RL (dB)=20 log|Zin−Z0Zin+Z0|,

where *Z*_in_ and *Z*_0_ are the impedances of the absorber and air, respectively; and *ε* and *μ* are the complex permittivity and permeability of the absorber, respectively; *f* is the frequency of EM waves; *d* is the thickness of the absorber; and *c* is the velocity of light. As shown in figure 7*a*, the calculated RLs of talc were less than −1.0 dB for all frequencies because of the low dielectric loss and magnetic loss for EM waves. As talc/NiTiO_3_ composite presented significantly higher dielectric loss tangents, it showed considerable absorption of EM waves. The maximum RLs of TN 450, TN 550 and TN 650 at a thickness of 2 mm were −0.94 dB at the frequency of 17.86 GHz, −1.10 dB at the frequency of 17.80 GHz and −2.90 dB at the frequency of 18.00 GHz, respectively. As discussed above, the values of permittivity and permeability of talc/NiTiO_3_ are quite lower than those of conventional carbon-based nanocomposites, such as carbon nanotubes (CNTs). CNTs have special helical structures and chiral properties, which will result in special EM performance. However, the lamella structure of talc weakened the quantum-limiting effects, so the RL may have been mainly induced by the dielectric loss of NiTiO_3_. On the other hand, it showed NiTiO_3_ with a morphology at nanoscale (figure 2*b–d*). It has been previously reported that nanoscale composites may cause a quantum tunnelling phenomenon. For the electrical conduction mechanism, the tunnelling transmission of the material can be calculated by the following equations:

3.3 $$\sigma_{\mathrm{DC}}^{\mathrm{tunnelling}}(V_{\mathrm{loc}},l)=\frac{2l}{{\left(2\pi \right)}^{2}}\int_{0}^{E_{F}}T(V_{\mathrm{loc}},E,l)\;\text{d}E,$$

where *E* is the total energy of the spilled particle, *E*_F_ is the Fermi energy of the fictitious medium, *l* is the dimer gap, *V*_loc_ is the local dielectric response and *T (V*_loc_*, E, l)* is the tunnelling (transmission) probability between the nanoparticles. From this perspective and figure 2*d*, it can be seen that NiTiO_3_ is dispersed on the talc lamella most uniformly, which will reduce the dimer gap to cause high *V*_loc_ and then Fowler–Nordheim tunnelling [38]. The magnetic loss can also be explained by the tunnelling effect, but may be weak. This suggests a possible physical reason that the EM wave absorption of talc/NiTiO_3_ may be affected by a combination of effects: the dielectric effect and the tunnelling effect.

The intrinsic wave impedances of these materials have been computed using the following equations:

3.4 $$\eta =\sqrt{\frac{\mu_{0}}{\varepsilon_{0}}}\sqrt{\frac{\mu^{\prime }}{\varepsilon^{\prime }}}.$$

**Figure 8.**
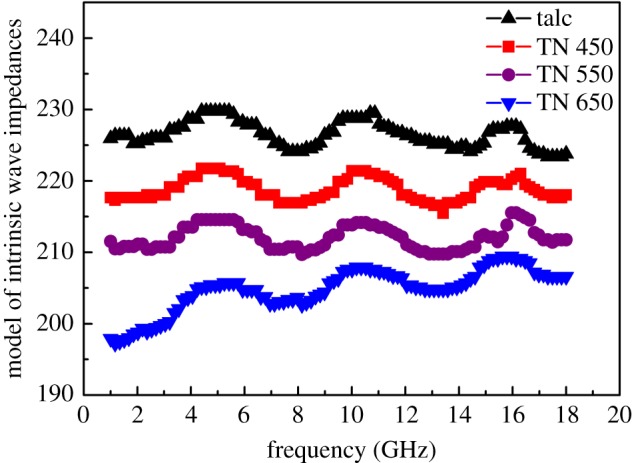
Frequency dependence of the intrinsic wave impedance of talc, TN 450, TN 550 and TN 650.

By inspection of measurements, it is worth noting that the relative permeability exhibits a real part very close (within the declared accuracy) to 1 and an imaginary part very close to 0. Therefore, we decided to neglect the effects on permeability, fixing *μ′* = 1. Therefore, higher electrical permittivity resulted in lower intrinsic wave impedance values. From figure 8, TN 650 showed the lowest value of intrinsic wave impendance versus frequency. By the above results, the trend of the electrical permittivity with the concentration of NiTiO_3_ was confirmed, with more NiTiO_3_ content deposited on the talc lamella with increase in calcined temperature. Previously, it was found that the EM properties of these composites can be tailored by controlling the content of the lossy materials, so a gradual matching is achieved through a high percentage of the absorber at the appropriate concentration range. In this work, TN 650 showed the highest EM performance even though with lowest impendance value [39].

**Figure 9.**
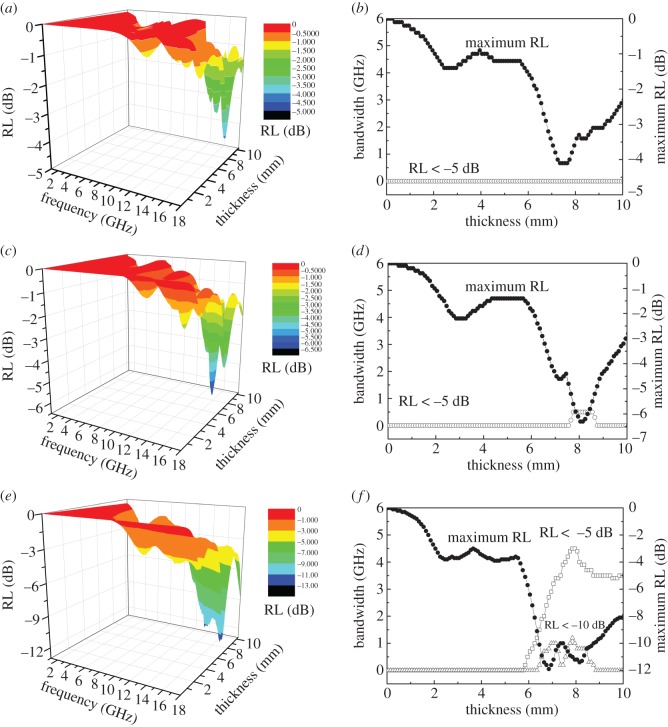
RL of talc/NiTiO_3_ composite as a function of thickness and the solid squares in pattern *b*, *d* and *f* are the frequencies with maximum RL as a function of thickness: (*a,b*) TN 450; (*c,d*) TN 550; (*e,f*) TN 650.

In addition, the RL of talc/NiTiO_3_ composite as a function of thickness was analysed to obtain the optimum thickness and frequency for EM absorption. As shown in figure 9, the maximum RL of talc/NiTiO_3_ composite increases with thickness. Thereafter, the maximum RL value of TN 450, TN 550 and TN 650 was about −4.07 dB at the thickness of 7.36 mm, −6.30 dB at the thickness of 8.10 mm and −11.94 dB at the thickness of 6.85 mm, respectively. There are two maximum bandwidth values of TN 650 in the bandwidth–thickness curve. The first maximum bandwidth for RL < −10 dB appears at the thickness of 6.3–7.3 mm, with a value of 1.05 GHz; the other appears at the thickness of 7.87 mm with a maximum bandwidth of 1.22 GHz for RL < −10 dB and 4.50 GHz for RL < −5 dB. It was also found that the maximum width of TN 550 was for RL < −5 dB at a value of 0.50 GHz at the thickness of 7.8–8.7 mm, and that of TN 450 was for RL < −5 dB at the thickness range of 0–10 mm. This verified that higher calcined temperature benefits talc/NiTiO_3_ composite to show higher permittivity and permeability to absorb EM waves, so TN 650 exhibits the optimum value for an EM wave absorber.

Because the increase in the thickness will significantly increase the weight of the absorber and thus restrict its applications, the optimum thickness of TN 650 for microwave absorption is 6.3–7.3 mm. The corresponding frequency at this thickness is 13–18 GHz, with a maximum RL of –11.94 dB, and a bandwidth of 1.04–3.41 GHz for RL < −5 dB and 0–1.05 GHz for RL < −10 dB.

When compared with other EM wave absorbing materials (table 2), due to the absence of magnetic components, talc/NiTiO_3_ composite showed lower absorption for EM waves than traditional EM wave absorbers, such as porous carbon fibre [40], porous Fe [41] and rice husk ash (SiC) [34]. However, the maximum RL, bandwidth < −5 dB and bandwidth < −10 dB are considerably higher than those for Fe pillared in halloysite [42] and montmorillonite blended with polymers [43]. In conclusion, talc/NiTiO_3_ is a considerable potential EM wave absorber.

**Table 2.** The comparison between the different EM wave-absorbing materials.

	thickness (mm)	bandwidth of RL < −5 dB (GHz)	bandwidth of RL < −10 dB (GHz)	maximum RL (dB)
TN 650	6.85	2.8	0.8	−11.94
porous carbon fibre [38]	2.9	/	10.9	−15.5
porous Fe [39]	2.0	4.6	2.2	−21.9
rice husk ash [34]	2.0	4.4	1.9	−12.5
halloysite–Fe [40]	2.0	/	/	−0.8
polypropylene/montmorillonite/ polypyrrole [41]	100	1.3	1.25	−60.0

## 4. Conclusion

In summary, we have successfully prepared talc/NiTiO_3_ composite using TNBT and nickel acetate to fabricate NiTiO_3_ and talc as the carrier. The morphology of talc exhibited a lamella structure; there were many nanoparticles of NiTiO_3_, with a diameter less than 20 nm, coated on the talc lamella. The thermal destruction occurred when it calcined, which increased the surface area from 2.51 to 79.05 m^2 ^g^−1^ at 650°C. The EM performance of talc/NiTiO_3_ composite was evaluated. Talc and talc/NiTiO_3_ composite showed considerable low permeability values caused by the absence of magnetic components. The presence of NiTiO_3_ increased dielectric loss and magnetic loss of talc. The calculated EM wave absorption of talc/NiTiO_3_ composite achieved a maximum RL of –11.94 dB at the thickness of 6.85 mm; the optimum thickness for microwave absorption is 6.3–7.3 mm, and the corresponding frequency at this thickness is 13–18 GHz. This study reveals a new approach for fabricating an EM absorber and broadening applications of both talc and NiTiO_3_ in EM absorption.

## Data accessibility

XRD data, BET and pore size data, and permittivity and permeability data are available at Dryad: http://dx.doi.org/10.5061/dryad.d1r5g [44].
